# Substernal oxyphil parathyroid adenoma producing PTHrP with hypercalcemia and normal PTH level

**DOI:** 10.1186/1477-7819-6-24

**Published:** 2008-02-21

**Authors:** Angela Gurrado, Andrea Marzullo, Germana Lissidini, Agostino Lippolis, Domenico Rubini, Gaetano Lastilla, Mario Testini

**Affiliations:** 1 Department of Applications in Surgery of Innovative Technologies, University Medical School of Bari, Italy; 2 Department of Pathology, University Medical School of Bari, Italy; 3 Department of Nuclear Medicine, University Medical School of Bari, Italy

## Abstract

**Background:**

Parathyroid adenoma is the most common cause of primary hyperparathyroidism. Preoperative serum calcium and intact-parathyroid hormone levels are the most useful diagnostic parameters that allow differentiating primary hyperparathyroidism from non-parathyroid-dependent hypercalcemia. Parathyroidectomy is the definitive treatment for primary hyperparathyroidism. Approximately 5% of patients who underwent parathyroidectomy present with persistent or recurrent hyperparathyroidism due to ectopic localization of the adenoma. Functioning oxyphil parathyroid adenoma is an uncommon histological form, seldom causing primary hyperparathyroidism. Parathyroid adenoma with hypercalcemia exhibiting normal parathyroid hormone level is rare. An incidence of 5% to 33% has been documented in the literature; no etiologic explanation has been given. In 1987, parathyroid-hormone-related peptide was isolated as a causative factor of humeral hypercalcemia of malignancy. The presence of parathyroid-hormone-related peptide in parathyroid tissue under normal and pathological conditions has been described in the literature; however, its role in causing hyperparathyroidism has not yet been defined.

**Case presentation:**

We present a case of persistent hypercalcemia with a normal level of intact-parathyroid hormone due to a substernal parathyroid adenoma, treated with radioguided parathyroidectomy. The final histological diagnosis was oxyphil adenoma, positive for parathyroid-hormone-related peptide antigens.

**Conclusion:**

In clinical practice, this atypical biochemical presentation of primary hyperparathyroidism should be considered in the differential diagnosis of hypercalcemia. The parathyroid-hormone-related peptide should be considered not only in the presence of malignancy.

## Background

The diagnosis of primary hyperparathyroidism (PHPT) is based on hypercalcemia and elevated levels of parathyroid hormone (PTH); additional laboratory hallmark features are hypophosphatemia and elevated urinary cyclic adenosine monophosphate (cAMP). The advent of intact-PTH immunoradiometric assay has further increased the reliability of results, enabling to distinguish PHPT from non-parathyroid-dependent hypercalcemia.

Eighty to 85% of patients with PHPT have a single parathyroid adenoma [1] that is ectopic in 5% to 10% of cases [2]. Ectopic parathyroid adenoma is a frequent cause of surgical failure and, therefore, several authors recommend preoperative imaging to localise the condition in patients with PHPT before initiating surgery. Thus, about 5% of patients who underwent parathyroidectomy present persistent or recurrent hyperparathyroidism [3].

Predominantly composed of chief cells, parathyroid adenomas are the most frequent cause of PHPT. Uncommonly, PHPT is caused by oxyphil adenoma, an infrequent histological form considered exclusively non-functioning until 1970 [4]. These adenomas consist of cells with abundant eosinophilic cytoplasm that correlates ultrastructurally with numerous mitochondria.

Presentation of hypercalcemia and normal PTH level due to parathyroid adenoma is infrequent; however, its incidence has been estimated to be between 5% and 33% [5].

In 1987, parathyroid-hormone-related peptid (PTHrP) has been isolated as a causative factor of humoral hypercalcemia of malignancy (HHM). The presence of PTHrP in parathyroid tissue has been described in the literature; however, its role in causing hyperparathyroidism has not yet been defined [6].

We present a case of persistent hypercalcemia with a normal level of intact-PTH due to a substernal oxyphil parathyroid adenoma positive for PTHrP, treated by radioguided parathyroidectomy.

## Case presentation

A 30-year-old man was referred to our Department of General Surgery for evaluation of persistent hypercalcemia. History revealed a 10-year history of constipation, recurrent episodes of renal colic, chronic fatigue, dyspepsia and hypercalcemia of unknown etiology. Eight months prior, in a different hospital, the patient underwent neck ultrasound that revealed a hypoechogenic mass located under the left thyroid lobe with an intense uptake during Tc-99m-sestamibi parathyroid scintigraphy. Subsequent surgical neck exploration had been unsuccessful.

Endocrinologic family history was uneventful; the patient's parents and two brothers had normal serum calcium levels and his one child was healthy. He did not take vitamin supplements, lithium or thiazide diuretics. Physical examination was negative, motor strength and reflexes were normal. Renal scan showed bilateral nephrolithiasis, and orthopantomography revealed signs of bone resorption at the jaw level.

Laboratory tests on admission showed hypercalcemia (12.7 mg/dL), hypophosphatemia (0.90 mg/dL), normal intact-PTH level (41.0 pg/mL) and an increase in 1,25-dihydroxycholecalciferol level (148.7 pg/mL). Urine biochemistry revealed elevated 24-hour urine calcium excretion (559 mg/24 h), phosphate clearance (57.8 mL/min) and urinary calcium to creatinine clearance 2 hours (0.30 mg/mg creatinine), whereas urinary cAMP was normal (3.9 nmol/100 mL glomerular filtrate). Thyroid and kidney function tests were within normal limits; tumor markers and blood tests for metabolic abnormalities were negative.

**Figure 1:**
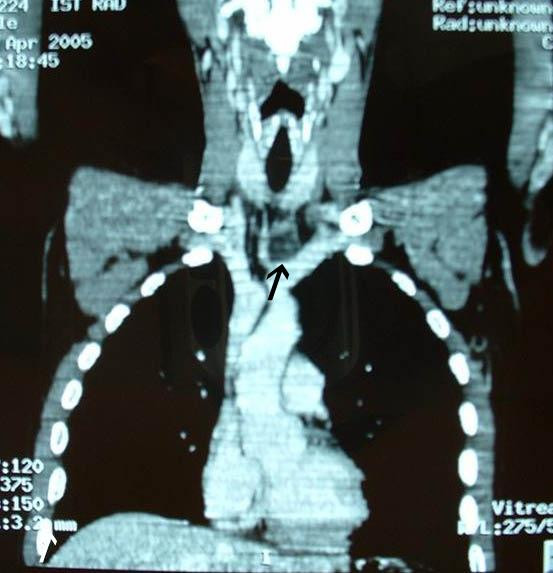
Multislice CT revealed an encapsulated mass of 3 cm in the upper anterior mediastinum behind the sternum-clavicular joints, with marked peripheral enhancement (arrow).

**Figure 2:**
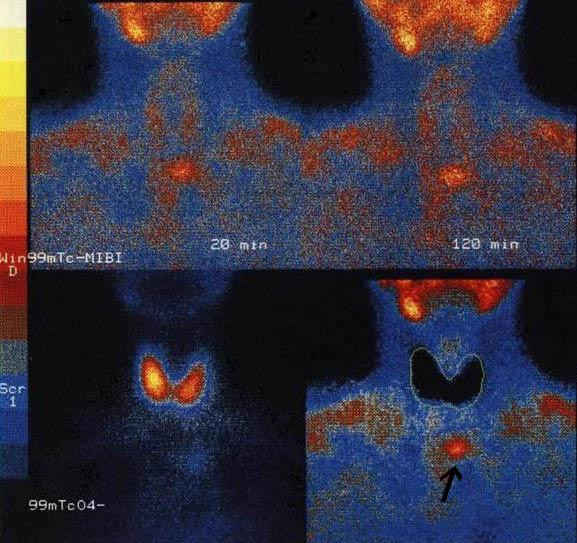
Tc-99m-sestamibi Tc-99m sodium pertechnetate substraction image showed an area of intense uptake below the inferior pole of the left thyroid lobe, in the upper mediastinum, in the left median position and normal thyroid with homogenous radiopharmaceutical uptake (arrow).

Neck ultrasound was not significant; thorax CT scan revealed an encapsulated mass of 3 cm in the upper anterior mediastinum located behind the sternoclavicular junction (Figure 1). Tc-99m sodium pertechnetate/Tc-99m-sestamibi parathyroid dual-phase scintigraphy with subtraction image technique revealed the mass to be an ectopic parathyroid adenoma at the level of the left sternoclavicular junction (Figure 2).

These data confirmed the hypothesis of parathyroid-dependent hypercalcemia. A radioguided adenoma resection through a collar reincision was performed. One hour prior to surgery, 50 MBq of Tc-99m – sestamibi was injected intravenously; in the operating room, the gamma probe (MR-100, 11C, Pol.hi.tech) was used to identify the following: *in vivo *localization of the cutaneous projection of the adenoma; ex vivo uptake of the 3.2 cm excised ectopic adenoma and, lastly, to confirm removal of the pathologic gland, on the "background" operated area. A biopsy of the right inferior parathyroid, though apparently normal, was performed. Intraoperative PTH was reduced by more than 50% compared to the normal preoperative level.

**Figure 3:**
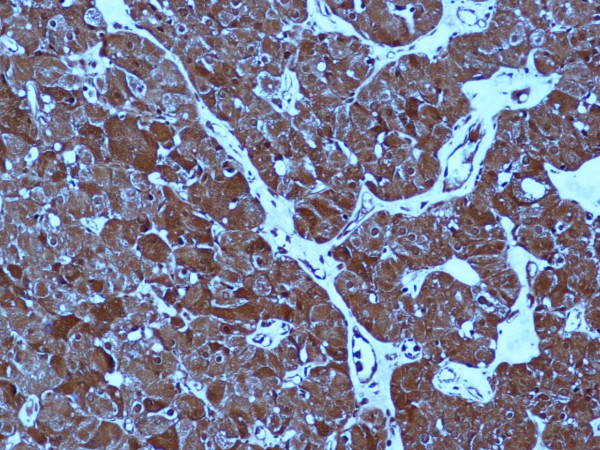
Strong and diffuse expression of PTHrP in the oxyphil adenoma (200 × original magnification).

Histologically, the tumor consisted of cords and small nests of cells with large granular eosinophilic cytoplasm with round to oval nuclei; the final diagnosis was oxyphil adenoma. Tumor cells were immunohistochemically positive for PTH (DakoCytomation; dilution 1:100; mouse) and PTHrP (Acris; 1:200; rabbit) antigens (Figure 3). The gland biopsy was normal.

Serum calcium level was normal in the immediate postoperative period (8.9 mg/dL) and 1, 6 and 12 months after surgery (8.6-8.9-8.4 mg/dL, respectively), with unchanged PTH within normal limits (11 pg/mL, day 1 after surgery; 9.4-13.0-17.0 pg/mL in 1, 6 and 12 months after surgery, respectively).

Recovery was uneventful and the patient was discharged 24 hours after the operation.

## Discussion

Hypercalcemia is one of the most common metabolic disorders and it may be generated by many different pathologic conditions. The most common categories are malignancy, PHPT and vitamin D-induced hypercalcemia; the less frequent include drug-induced conditions (*eg*, lithium, thiazide diuretics), immobilization, tuberculosis, sarcoidosis and rhabdomyolysis.

Identifying disease etiology is very important, since, subsequent management differs according to pathology. Although a detailed personal and family history, physical examination, and laboratory analyses can differentiate the causes in most cases, hypercalcemia often remains a challenging condition for clinicians. PTH level is the classic discriminator between parathyroid disease-dependent hypercalcemia and others, whereas PTHrP is the most useful analytical method in HHM.

The most frequent cause of PHPT is a solitary adenoma composed mainly of chief cells producing PTH. In contrast, oxyphil adenoma meets the following criteria: 1) consists of at least 90% oxyphil cells; 2) histologically normal excision or biopsy of a second gland excluding the possibility of parathyroid hyperplasia; and 3) immediate postoperative normalization of hypercalcemia [7]. In this reported case, all the criteria were met.

The literature review reports 142 cases of oxyphil parathyroid adenomas [8-10]. The ability of this histological form to secrete PTH has been demonstrated during the last 30 years [11] ; in fact, hypercalcemia was associated to secretion of PTH due to oxyphil adenomas in only 124 of 142 reported cases [8,10,11].

Since 1976, approximately 140 cases of hypercalcemia and normal PTH levels due to parathyroid adenoma have been reported in the literature [12-15] ; however, no correlation between the atypical biochemical presentation, histological features and causative factors has been demonstrated. In this regard, Hollemberg *et al*. [14] and Perez *et al*. [13] proposed the following theoretical explanations to the mechanism of inappropriately low PTH level in the PHPT cases observed: a circulating inhibitor of PTH; the pulsatile secretion of the hormone; an abnormal PTH molecule with increased biologic activity; increased peripheral tissue sensitivity to normal PTH; or the presence of another mediator of hypercalcemia (eg, PTHrP). None of these hypotheses have been verified. Lafferty *et al*. [12] emphasized that PHPT cannot always be ruled out because of a low-normal serum intact or biointact PTH and suggested that repeated PTH measurements after serum dilution in suspected cases of PHPT with low-normal PTH levels may be a useful method for detecting atypical forms of PTH.

PTHrP is a factor responsible for malignancies associated with hypercalcemia such as lung cancer, breast carcinoma, pancreatic endocrine tumors, renal cell carcinoma; esophageal squamosous cell carcinoma, pheochromocytoma and hematological neoplasia. PTHrP has been demonstrated in adult human parathyroid tissue under normal and pathological conditions; however, the role of this peptid secreted in 12 reported cases of oxyphil parathyroid adenoma associated with hypercalcemia has not yet been defined [9,10]. In fact, in 11 cases, the PTH level has not been reported [9], and in one, hypercalcemia depended on simultaneous hypersecretion of PTH [10].

Nevertheless, these studies have established the correlation of PTHrP expression with the age-related metaplastic change of parathyroid cells into the oxyphil phenotype, through a paracrine/autocrine regulatory mechanism [9,10].

To our knowledge, the association of immunohistochemical positivity for PTHrP-antigens with normal PTH levels, the immediate postoperative normalization of hypercalcemia, and the histological features of parathyroid adenoma have not been previously reported.

Moreover, technetium Tc-99m sestamibi imaging techniques have played a very important role in defining the preoperative localization in this case study. The predominance of oxyphil cells within an adenoma actually increases Tc-99m-sestamibi scan sensitivity in a statistically significant manner, since sestamibi uptake likely increases in more metabolically active lesions and when the preoperative calcium level is ≥ 9.5 mg/dL so as to reduce the risks of bilateral surgical neck exploration and persistent PHPT [8].

## Conclusion

In light of the reported data, PTHrP plays a critical role in defining the pathogenesis of atypical hypercalcemia due to parathyroid adenoma without hypersecretion of PTH. In clinical practice, this biochemical presentation should be considered in the differential diagnosis of hypercalcemia, and serum PTHrP should be assessed not only in the presence of malignancy. Additional studies with larger populations are necessary to associate the various clinical and biochemical presentations of PHPT with preoperative diagnostic flow-charts.

## Competing interests

The author(s) declare that they have no competing interests.

## Authors' contributions

AGcontributed substantially to manuscript conception and drafted the manuscript. AMperformed histopathological and immunohistochemical analyses. GeLwas responsible for critical revision of scientific content. ALrevised the manuscript critically for important intellectual content. GaLcontributed to the pathology content DRperformed the gamma probe examination. MTthe surgeon; approved the final version of the manuscript for publication. All authors read and approved the final version of the manuscript.
